# Modulation of Toll-like receptor signaling by intestinal bacteria in colorectal cancer

**DOI:** 10.3389/fcimb.2026.1963456

**Published:** 2026-10-12

**Authors:** Shuang Wang, Dongliang Chen, Dawei Yang, Lei Gao, Yifan Zhang

**Affiliations:** 1 Zhong Yuan Academy of Biological Medicine, Liaocheng People’s Hospital, Liaocheng, China; 2 Center for Innovation and Technology Transfer, Liaocheng People’s Hospital, Liaocheng, China

**Keywords:** bacterial product, colorectal cancer, host-microbiome interactions, intestinal bacteria, Toll-like receptor, tumor microenvironment

## Abstract

The gut microbiota contributes to intestinal homeostasis through dynamic interactions with epithelial, immune and stromal cells. Disruption of the balance, often termed dysbiosis, has been associated with colorectal cancer (CRC). At the same time, selected commensal bacteria have shown protective effects. Toll-like receptors (TLRs) provide an important molecular interface through which host cells detect bacterial components and initiate cellular responses. This Mini Review examines current evidence that intestinal bacteria and their products modulate TLR signaling in CRC, with effects that may promote or restrain tumor development. The reported mechanisms involve CRC cells as well as immune and stromal compartments within the tumor microenvironment. The available evidence does not support assigning a fixed biological effect to a bacterial species or TLR. Instead, the reported outcomes appear to vary with the nature of the bacterial stimulus, the responding cell and the disease setting. Resolving this context dependence may clarify how host cells translate bacterial stimuli through TLRs into responses that influence CRC progression and treatment response.

## 1 Introduction

In 2024, colorectal cancer (CRC) was estimated to account for more than 2.0 million new cases and 918,000 deaths worldwide, making it the third most commonly diagnosed cancer and the second leading cause of cancer death (Sung et al., 2026). Incidence varies geographically and is rising among adults younger than 50 years in many populations (Lee et al., 2026). CRC develops through the accumulation of genetic and epigenetic alterations (Li et al., 2024). Disease risk is influenced by aging, chronic intestinal inflammation and modifiable exposures, including diet, excess adiposity, physical inactivity, alcohol consumption and smoking (Murphy and Zaki, 2024). Several of these exposures also change intestinal microbial composition or function, raising the possibility that the microbiota mediates some of their effects on CRC risk (Song et al., 2020).

Meta-analyses of fecal metagenomes have identified reproducible CRC-associated taxonomic and functional signatures across populations (Thomas et al., 2019; Wirbel et al., 2019). Microbial and metabolite profiles nevertheless vary across adenomas and CRC stages (Yachida et al., 2019). Recent pooled analyses confirmed shared signals but revealed variation by stage, tumor location, geography and bacterial lineage (Piccinno et al., 2025; Pekel et al., 2026). Tissue studies likewise found distinct profiles across non-neoplastic, polyp and CRC tissues, although associations with anatomical location, molecular subtype and prognosis differed between cohorts (Shi et al., 2026; Xiang et al., 2026). Transit time, fecal inflammation, adiposity and control selection can influence reported associations, some of which weaken after quantitative profiling and covariate adjustment (Tito et al., 2024). Despite these variations, tissue and fecal profiling provide valuable information for identifying CRC-associated microbial signatures.

Direct evidence linking a microbial product to human CRC remains uncommon, with colibactin as one example. Repeated exposure of human intestinal organoids to colibactin-producing *pks*^+^ *Escherichia coli* generated mutational signatures also detected in some CRC genomes (Pleguezuelos-Manzano et al., 2020). A colibactin-associated DNA-damage pattern was enriched at CRC mutational hotspots (Dziubańska-Kusibab et al., 2020), and SBS88 and ID18 were linked to early-onset CRC and shown to arise early in tumor development (Díaz-Gay et al., 2025). These footprints record past genotoxin exposure rather than indicating current carriage of the producer. For most CRC-associated bacteria, causal attribution and the relevant host-response pathways remain less secure (Wong and Yu, 2023). Proposed mechanisms extend beyond genotoxicity to disrupted epithelial barrier function and modulation of inflammatory and immune responses (González et al., 2024). Some of these effects may depend on how bacterial components and products are sensed by host cells.

Toll-like receptors (TLRs) enable host cells to recognize conserved microbial structures known as pathogen-associated molecular patterns (PAMPs). Surface and endosomal TLRs recognize bacterial lipoproteins, lipopolysaccharide (LPS), flagellin and nucleic acids. The resulting signaling regulates innate and adaptive immunity, tissue repair and inflammation, and its dysregulation may contribute to CRC development (Burgueño and Abreu, 2020; Fitzgerald and Kagan, 2020). This Mini Review examines how intestinal bacteria engage TLR signaling in CRC, emphasizing tumor-promoting and protective mechanisms, unresolved questions and translational challenges.

## 2 Context-dependent TLR signaling in colorectal tumorigenesis

In the intestine, TLR signaling contributes to normal mucosal function rather than being intrinsically tumor-promoting. The colonic epithelium is continually exposed to gut microbiota, and basal recognition of the resident commensals by TLRs supports epithelial homeostasis, repair and resistance to injury (Rakoff-Nahoum et al., 2004). Barrier disruption exposes epithelial and lamina propria cells to microbial products, promoting inflammatory circuits that can support tumor growth (Grivennikov et al., 2012; Slowicka et al., 2020). With the exception of TLR3, TLRs use MyD88-dependent signaling. Endocytosed TLR4 can additionally signal through TRAM and TRIF, while TLR3 signals exclusively through TRIF. MyD88 and TRIF activate overlapping NF-κB and mitogen-activated protein kinase pathways, and TRIF also promotes IRF3-dependent type I interferon responses (Kawai and Akira, 2010). Notably, receptors such as IL-1R1 and IL-18R also signal through MyD88 (Adachi et al., 1998), so a MyD88-dependent phenotype is not necessarily mediated by TLRs.

The most extensive receptor-specific evidence in intestinal tumorigenesis concerns TLR4. The azoxymethane/dextran sulfate sodium (AOM/DSS) model combines carcinogen exposure with chemically induced colitis to study inflammation-associated colorectal tumorigenesis. In this model, global *Tlr4* deficiency reduced tumor burden, with corresponding decreases in COX-2 expression, PGE_2_ production, amphiregulin abundance and EGFR phosphorylation (Fukata et al., 2007). *Tlr4* deficiency also reduced tumor burden in *Apc^Min/+^* mice, which carry a germline mutation in the tumor-suppressor gene *Apc* and develop spontaneous intestinal adenomas (Shi et al., 2020b). Villin-TLR4 mice express a constitutively active form of TLR4 in the intestinal epithelium. Constitutive epithelial TLR4 expression expanded *Lgr5*-positive cells, increased β-catenin activity and promoted AOM-induced tumorigenesis (Santaolalla et al., 2013). Subsequent work showed that epithelial TLR4 induced DUOX2-dependent hydrogen peroxide production. Loss of epithelial DUOX activity and germ-free housing each reduced tumorigenesis in villin-TLR4 mice. Mucosal microbiota from these mice increased epithelial hydrogen peroxide production and AOM/DSS-associated tumor burden after transfer to wild-type germ-free recipients (Burgueño et al., 2021). Host metabolism may also modify microbiota-driven TLR4 responses. In mice, ceramide-induced dysbiosis increased tumor burden through TLR4-dependent β-catenin activation and SOAT1-mediated cholesterol esterification (Zhu et al., 2022). In a separate study, FADS1-dependent arachidonic acid production favored Gram-negative bacteria that stimulated TLR4–MyD88-dependent PGE_2_ synthesis and promoted tumor growth in AOM/DSS and intestine-specific *Apc*-deficient mice (Xu et al., 2023).

TLR4 signaling during intestinal tumorigenesis involves several cellular compartments. Reciprocal bone marrow chimeras, used to assess the relative contributions of TLR4 in bone-marrow-derived cells and recipient tissues, supported a role for TLR4 in recipient colonic epithelial cells in recruiting COX-2-expressing macrophages and increasing dysplasia in the AOM/DSS model (Fukata et al., 2009). During early AOM/DSS tumorigenesis, epithelial LPS stimulation induced CCL2 and recruited monocyte-like macrophages. LPS-activated macrophages produced IL-1β that supported Th17-cell expansion, and TLR4 inhibition attenuated these responses (Yang et al., 2021). Conditional *Tlr4* deletion in collagen VI-positive intestinal mesenchymal cells, including cancer-associated fibroblasts (CAFs), reduced tumorigenesis in *Apc^Min/+^* mice, but did not reduce AOM/DSS tumor formation (Koliaraki et al., 2019). Together, these findings imply that tumor-promoting TLR4 activity is a multicellular process, with epithelial, immune and stromal responses contributing differently across disease settings.

Loss-of-function studies suggest protection by TLR2 and TLR6 in inflammation-associated models, but TLR2–TLR1 and TLR2–TLR6 agonists produced different effects in CRC cells. Global *Tlr2* or *Tlr6* deficiency increased AOM/DSS tumorigenesis (Lowe et al., 2010; Kim et al., 2020). The TLR1/TLR2 agonist Pam3Cys increased CRC-cell proliferation, while *TLR2* deletion reduced proliferation and xenograft growth (Liu et al., 2018). The TLR2/TLR6 agonist Pam2CSK4 instead restrained CRC-cell growth and invasion, and *TLR6* knockdown enhanced xenograft growth (Ma et al., 2024). Studies of TLR3 likewise distinguish receptor function from agonist treatment. *Tlr3* deficiency increased tumor burden in AOM/DSS models, yet treatment with poly(I:C) did not suppress tumorigenesis (Chung et al., 2024). A protective genetic phenotype thus does not necessarily predict therapeutic benefit from receptor agonism. The effects of targeting TLR signaling may depend on treatment timing. Peritumoral flagellin suppressed DLD-1 xenograft growth through TLR5 when treatment began early after implantation but not after tumors were established (Rhee et al., 2008). In the AOM/DSS model, TAK-242 administered during DSS-associated inflammation reduced subsequent tumor burden. The authors reported no detectable change in tumor number or size when treatment was given from weeks 6 to 9 during an advanced tumor phase (Pastille et al., 2021). These studies also suggest that responses to TLR-directed interventions may differ between early tumor development and established disease.

Human studies associate tumor TLR expression with microbial and immune features but rarely resolve receptor–ligand relationships. TLR2, TLR4 and TLR5 expression differed in adenomas and CRC and correlated with selected fecal bacteria in a small case–control study (Aghdaei et al., 2022). High TLR2 and TLR4 expression were modestly associated with denser intratumoral CD3^+^ and CD8^+^ lymphocyte infiltrates (Beilmann-Lehtonen et al., 2023). Two nonsynonymous *TLR5* variants were associated in opposite directions with CRC-specific survival and showed differential responses to flagellin and intestinal bacteria (Klimosch et al., 2013). Associations between TLR4 expression and CRC outcome have been inconsistent and may vary with disease stage (Crame et al., 2023).

These observations establish clinical associations, but receptor abundance does not necessarily indicate microbial signal, responding cell, or pathway activity. Against this clinical background, studies of defined bacteria and bacterial products provide a more direct basis for examining TLR-dependent effects in CRC.

## 3 Tumor-promoting bacterial modulation of TLR signaling in CRC

Studies suggest that CRC-associated bacteria may contribute to tumorigenesis through TLR signaling in cancer cells and the surrounding microenvironment. Reported effects include changes in cell proliferation, plasticity and metabolism, together with immune and stromal responses that support dissemination or chemoresistance. Evidence ranges from receptor perturbation in cultured cells to receptor-deficient mouse models. Selected proposed mechanisms are summarized in **Figure 1**.

**Figure 1:**
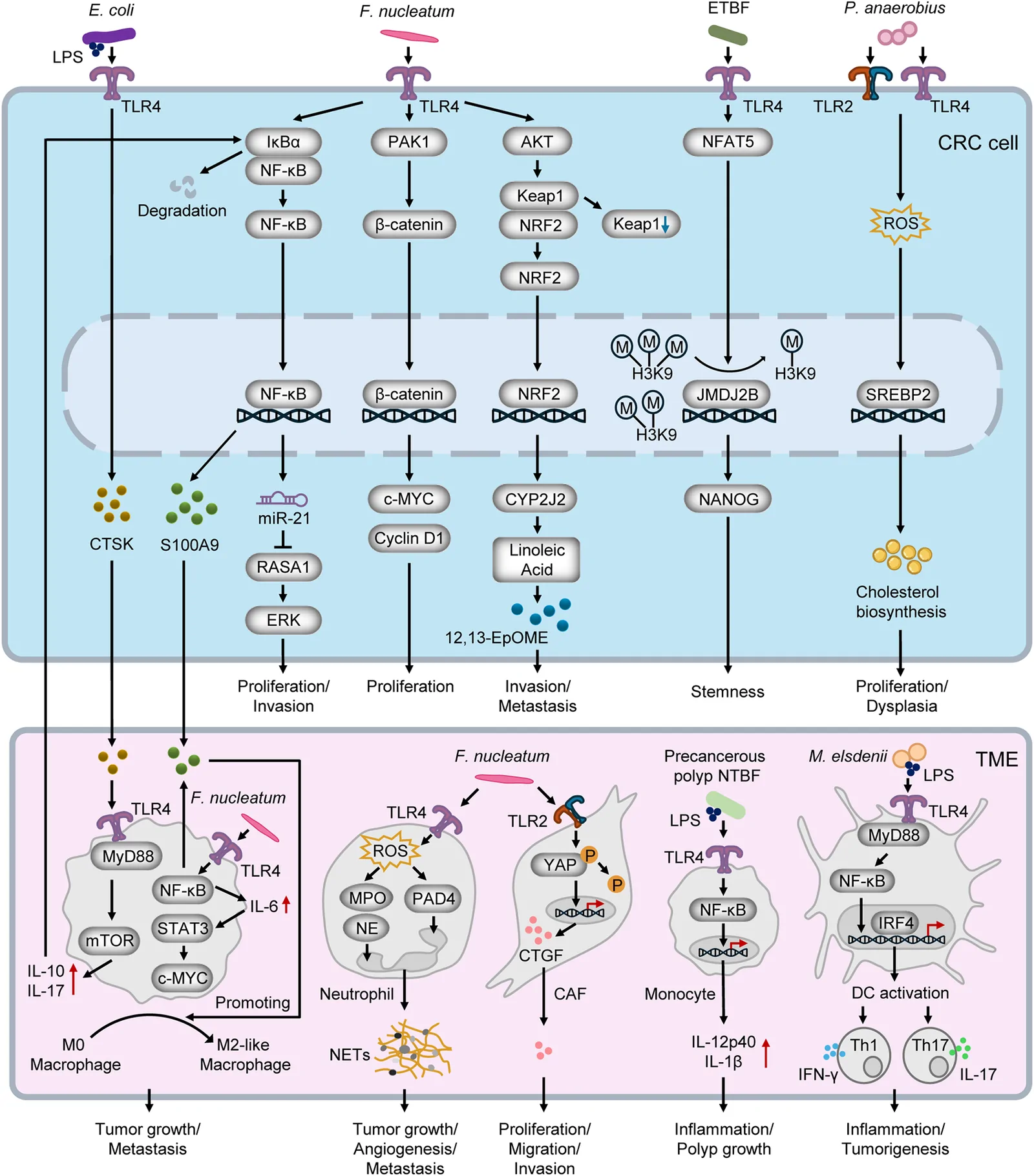
Proposed mechanisms by which intestinal bacteria may promote colorectal cancer through TLR signaling. The upper panel summarizes proposed responses in CRC cells, and the lower panel depicts responses in immune and stromal compartments of the tumor microenvironment. Depending on the bacterial strain or product and the responding host cell, TLR-associated signaling may influence proliferative, stemness, metabolic, inflammatory and paracrine programs linked to tumor growth and dissemination. Arrows indicate proposed functional relationships and do not necessarily denote direct ligand–receptor binding. Most mechanisms are based on preclinical studies and should be interpreted as strain-, cell- and context-dependent. CAF, cancer-associated fibroblast; CRC, colorectal cancer; DC, dendritic cell; EMT, epithelial–mesenchymal transition; ETBF, enterotoxigenic *Bacteroides fragilis*; LPS, lipopolysaccharide; NETs, neutrophil extracellular traps; NTBF, non-toxigenic *Bacteroides fragilis*; TME, tumor microenvironment.

Certain intestinal bacteria may promote colorectal tumorigenesis by activating TLR signaling in epithelial cells. *Fusobacterium nucleatum*, an oral anaerobe repeatedly enriched in CRC tissues (Castellarin et al., 2012; Kostic et al., 2012), activated β-catenin signaling via a TLR4–PAK1 cascade in CRC cells (Chen et al., 2017). In antibiotic-treated *Apc^Min/+^* mice, *F. nucleatum* increased intestinal tumor burden, while TLR4 inhibition reduced tumor growth and PAK1–β-catenin activation (Wu et al., 2018). Hence, TLR4 may amplify β-catenin-driven tumorigenesis. Interestingly, *F. nucleatum* induced oncogenic miR-21 through TLR4 signaling in CRC cells. miR-21 directly repressed RASA1 and was associated with MAPK activation. Blocking miR-21 restored RASA1 expression and attenuated the bacterium-induced increases in proliferation and invasion, revealing post-transcriptional regulation downstream of TLR4 (Yang et al., 2017). A distinct stemness program was reported for enterotoxigenic *Bacteroides fragilis* (ETBF). In CRC cells, TLR4-dependent induction of NFAT5 increased JMJD2B, which reduced repressive H3K9 trimethylation at the *NANOG* promoter and increased NANOG expression. Knockdown of *TLR4* or *JMJD2B* attenuated ETBF-induced tumorsphere formation and xenograft growth (Liu et al., 2020).

TLR signaling has also been linked to host lipid metabolism. *Peptostreptococcus anaerobius* increased dysplasia in antibiotic-depleted, AOM-treated mice. In colonic epithelial cells, it promoted reactive oxygen species (ROS) production, SREBP2-dependent cholesterol synthesis and proliferation through TLR2- and TLR4-dependent signaling (Tsoi et al., 2017). *F. nucleatum* increased CYP2J2-derived 12,13-epoxyoctadecenoic acid (12,13-EpOME) through TLR4-dependent NRF2 signaling. 12,13-EpOME promoted epithelial–mesenchymal transition and liver colonization (Kong et al., 2021).

Beyond the epithelium, bacteria can activate TLR signaling in immune and stromal cells to reshape the tumor microenvironment. In antibiotic-treated mice, oral *E. coli* administration increased intratumoral LPS levels, primary tumor growth and liver metastasis. LPS induced cathepsin K (CTSK) in CRC cells, and this tumor-derived protease was reported to engage macrophage TLR4 and promote an M2-like phenotype (Li et al., 2019). *F. nucleatum* also induced tumor-supportive macrophage programs associated with M2 polarization. In *Apc^Min/+^* mice, oral *F. nucleatum* increased M2-like macrophage accumulation and intestinal tumor burden. Both effects were reduced by TLR4 inhibition, while experiments in cultured macrophages implicated IL-6–STAT3–c-MYC signaling (Chen et al., 2018). In a separate study, *F. nucleatum* induced S100A9 in macrophages and CRC cells through TLR4–NF-κB signaling. *S100A9* silencing attenuated M2-like polarization and partly reduced the ability of *F. nucleatum*-exposed macrophages to promote CRC-cell proliferation, migration and xenograft growth (Hu et al., 2021).

*Megasphaera elsdenii* and its LPS activated dendritic cells through TLR4 signaling involving NF-κB and IRF4. Dendritic cells primed by the bacterium promoted Th1 and Th17 responses. In AOM/DSS-treated mice, *M. elsdenii* increased tumor burden in wild-type but not *Tlr4*-deficient mice (Hou et al., 2025).

Neutrophil extracellular traps (NETs) have been shown to support CRC progression (Yazdani et al., 2019; Shang et al., 2020). *F. nucleatum* induced NETs through TLR4–ROS and parallel NOD1/2 signaling, and DNase I attenuated the associated tumor growth and metastasis (Kong et al., 2023). An *E. coli* K-12 strain likewise promoted NET-dependent liver metastasis. Combined TLR2 and TLR4 inhibition partly reduced NET release, while NOD1/2 blockade had a larger effect (Pan et al., 2026). NET formation can arise downstream of distinct pattern-recognition pathways (Wang et al., 2024), although their relative contribution appears to vary with the bacterial stimulus.

Recently, *F. nucleatum* was reported to signal through TLR2 in CAFs, promoting YAP nuclear localization and connective tissue growth factor (CTGF) production that supported CRC-cell proliferation and migration (Wang et al., 2025). Separate work showed that *F. nucleatum* exposure induced an inflammatory CAF phenotype, which was partially mediated by TLR4 signaling and enhanced CRC-cell invasion (Karta et al., 2025).

TLR4 signaling may also affect chemotherapy response. *F. nucleatum* reduced miR-18a* and miR-4802 through *TLR4*–*MYD88* signaling, increasing their respective autophagy targets ULK1 and ATG7. Restoring either miRNA or silencing *TLR4* or *MYD88* attenuated autophagy and resistance to 5-fluorouracil and oxaliplatin in CRC cells and xenografts (Yu et al., 2017). The bacterium also induced BIRC3, an inhibitor of apoptosis, through TLR4–NF-κB signaling. Silencing *BIRC3* restored 5-fluorouracil-induced apoptosis and partly reduced chemoresistance in xenografts (Zhang et al., 2019). More recently, *F. nucleatum* induced the long non-coding RNA *PVT1* through TLR4–NF-κB signaling in CRC cells. *PVT1* stabilized ATAD3A and limited cell death caused by oxaliplatin-induced endoplasmic reticulum stress. *PVT1* depletion partly restored oxaliplatin sensitivity in CRC cells and xenografts (Gao et al., 2025). LPS from *Klebsiella pneumoniae* or *E. coli* activated TLR4 signaling in macrophages, eliciting a paracrine response that suppressed p53 activity and reduced chemotherapy sensitivity in CRC cells (Fragkoulis et al., 2026).

## 4 Protective bacterial modulation of TLR signaling in CRC

The protective effects attributed to intestinal bacteria are not simply the inverse of microbe-associated tumor promotion, nor do they invariably arise from TLR activation. The label “tumor-suppressive bacterium” describes an observed phenotype rather than an intrinsic property of a bacterial species or a defined mode of action. **Figure 2** summarizes selected protective mechanisms of intestinal bacteria involving TLR signaling.

**Figure 2:**
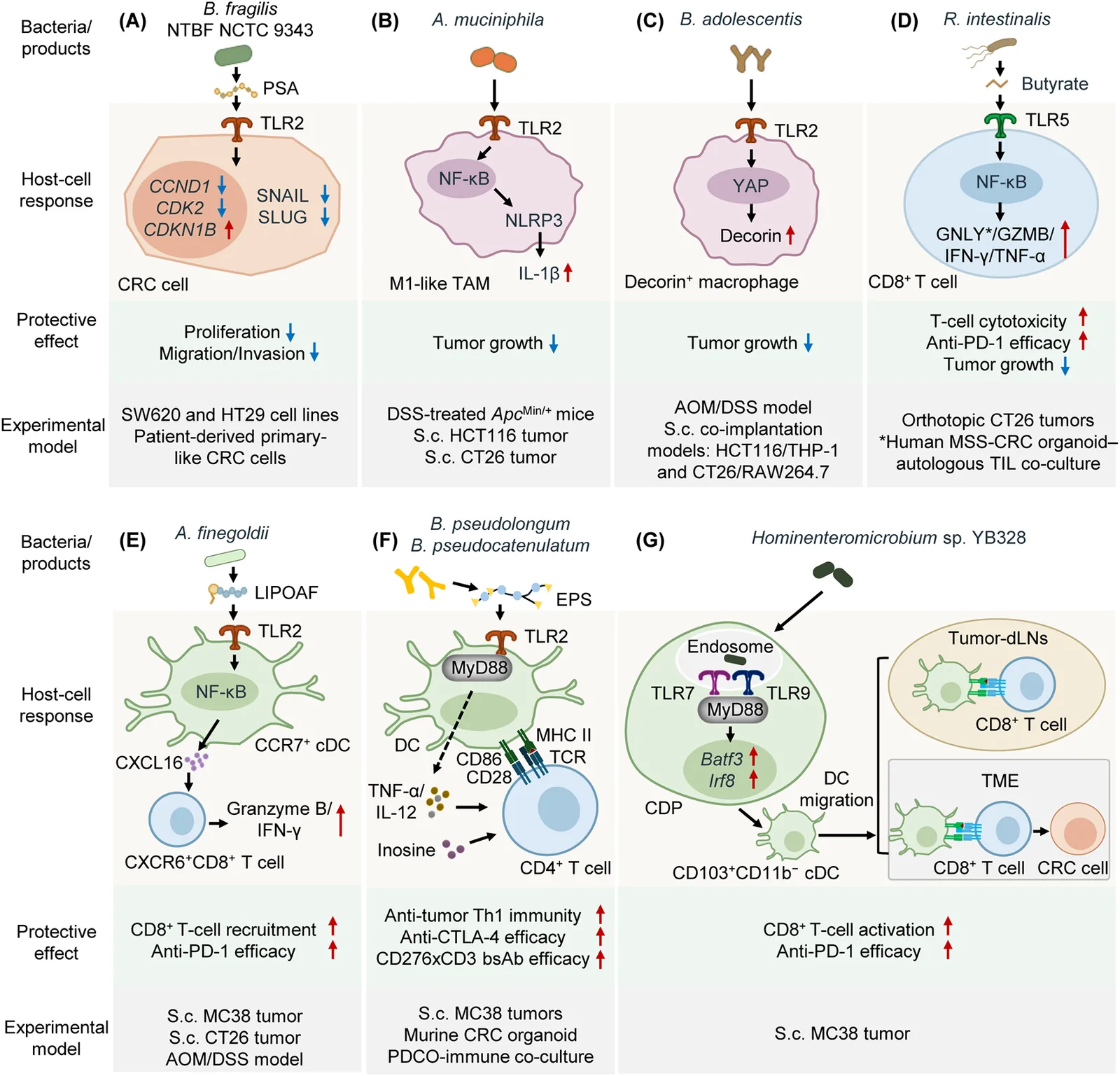
Selected protective mechanisms of intestinal bacteria involving TLR signaling in colorectal cancer. **(A)** NTBF PSA-associated TLR2 responses, including changes in cell-cycle gene expression and reduced CRC-cell proliferation, migration and invasion. **(B)** *A. muciniphila* induces TLR2–NLRP3 signaling, M1-like macrophage polarization and IL-1β release. **(C)** *B. adolescentis* induces decorin-positive macrophages involving TLR2–YAP signaling and suppression of tumor growth. **(D)** Butyrate from *R. intestinalis* enhances CD8^+^ T-cell cytotoxicity and anti-PD-1 efficacy involving TLR5–NF-κB signaling. Human organoid–autologous TIL cocultures received supplied sodium butyrate. GNLY* denotes findings from human experiments, not the mouse models. **(E)** *A. finegoldii*-derived LIPOAF stimulates dendritic cell TLR2 signaling and CXCL16 production, supporting CXCR6^+^ CD8^+^ T-cell recruitment. The listed CT26 and AOM/DSS experiments used the whole bacterium. **(F)** Bifidobacterial EPS activates dendritic cell TLR2–MyD88 signaling and, together with inosine, supports Th1 responses and anti-CTLA-4 efficacy. *B. pseudolongum* EPS also enhances CD276 × CD3 bsAb-mediated killing of human CRC organoids. **(G)** Proposed YB328-associated cDC differentiation involving TLR7/9–MyD88 signaling, with dendritic cell migration to tumor-draining lymph nodes and tumors supporting CD8^+^ T-cell responses and anti-PD-1 efficacy. AOM/DSS, azoxymethane/dextran sulfate sodium; bsAb, bispecific antibody; cDC, conventional dendritic cell; CDP, common dendritic cell progenitor; CRC, colorectal cancer; CTLA-4, cytotoxic T-lymphocyte-associated protein 4; DC, dendritic cell; dLN, draining lymph node; EPS, exopolysaccharide; GNLY, granulysin; GZMB, granzyme B; LIPOAF, lipoprotein derived from *A. finegoldii*; MHC, major histocompatibility complex; MSS, microsatellite stable; NTBF, non-toxigenic *B. fragilis*; PD-1, programmed cell death protein 1; PDCO, patient-derived cancer organoid; PSA, polysaccharide A; s.c., subcutaneous; TAM, tumor-associated macrophage; TCR, T-cell receptor; Th1, T helper 1 cell; TIL, tumor-infiltrating lymphocyte; TLR, Toll-like receptor; TME, tumor microenvironment.

Non-toxigenic *Bacteroides fragilis* (NTBF) illustrates why strain identity and host setting matter. In the AOM/DSS model, the NTBF strain NCTC 9343 inhibited tumor formation, and protection was lost with a polysaccharide A (PSA)-deficient mutant or in *Tlr2*-deficient mice (Lee et al., 2018). In human CRC cells, PSA also limited proliferation, migration and invasion, accompanied by changes in cell-cycle and EMT-associated markers. *TLR2* knockdown attenuated these effects (Sittipo et al., 2018). In mono-colonized gnotobiotic mice, NCTC 9343 reduced tumor incidence in wild-type but not *Tlr4*-deficient hosts (Lee et al., 2021). This profile should not be generalized to NTBF as a group. *bft*-negative NTBF isolates associated with precancerous polyps were enriched in genes involved in LPS biosynthesis, and preferentially activated TLR4 and elicited a pro-inflammatory cytokine profile that may favor polyp growth (Kordahi et al., 2021). These studies support evaluating specific bacterial strains, along with their characterized products or components in relevant disease settings.

Protective bacterial sensing can also operate through immune cells within the tumor microenvironment. In MC38-bearing mice, *Hominenteromicrobium* strain YB328 promoted MyD88- and TLR7/TLR9-dependent generation of intestinal CD103^+^CD11b^-^ conventional dendritic cells. Photoconversion tracing showed that these cells migrated to tumors, where they supported tumor-specific CD8^+^ T-cell responses and enhanced the response to PD-1 blockade (Lin et al., 2025). *Bifidobacterium adolescentis* reduced AOM/DSS-induced tumorigenesis and increased a decorin-positive macrophage population identified by single-cell RNA sequencing, with subsequent experiments implicating TLR2–YAP signaling in decorin induction. Knockdown of *Dcn* weakened the ability of *B. adolescentis*-treated macrophages to suppress tumor growth in a co-implantation model with CT26 cells (Lin et al., 2023). *Lacticaseibacillus rhamnosus* GG administered after AOM/DSS tumors had formed reduced tumor burden through a CD8^+^ T-cell-dependent response. Separate experiments showed that its activation of dendritic cells and expansion of colonic CD8^+^ T cells required TLR2 (Owens et al., 2021).

*Akkermansia muciniphila* also activated TLR2 to induce NLRP3-dependent M1-like tumor-associated macrophage (TAM) polarization and IL-1β release, thereby exerting antitumor effects (Fan et al., 2021). The outer-membrane protein Amuc_1100 activated a human TLR2 reporter *in vitro*, and structural work demonstrated its binding to recombinant human TLR2 (Ottman et al., 2017; Wang et al., 2020a). Purified Amuc_1100 enhanced IL-2-mediated control of CT26 tumors, and pharmacological TLR1–TLR2 blockade attenuated the response (Shi et al., 2020a). Amuc_1100 also reduced colitis-associated tumorigenesis through modulation of cytotoxic T lymphocytes (Wang et al., 2020b). Consistently, the C-terminal fragment of Amuc_1100, termed Amuc_C, was proposed as a novel TLR2 agonist and suppressed CT26 tumor growth, accompanied by increased CD8^+^ T cells and M1-like macrophages (Chi et al., 2025).

Immune modulation involving TLR signaling has also been reported for other defined bacterial products. Exopolysaccharides from *Bifidobacterium pseudolongum* and *Bifidobacterium pseudocatenulatum* activated TLR2–MyD88 signaling in dendritic cells. Together with inosine and anti-CTLA-4, these preparations reduced MC38 tumor growth in germ-free mice (Silva de Oliveira et al., 2025). The *Alistipes finegoldii* lipoprotein LIPOAF acted through TLR2 on CCR7-positive conventional dendritic cells and induced CXCL16, recruiting CXCR6-positive CD8^+^ T cells and improving checkpoint blockade in MC38 tumors (Wu et al., 2025). *Roseburia intestinalis*-derived butyrate is reported to bind TLR5 on CD8^+^ T cells to boost cytotoxicity and enhance anti-PD-1 efficacy in CRC mouse models (Kang et al., 2023). In autologous microsatellite-stable CRC organoid–tumor-infiltrating lymphocyte (TIL) cocultures, butyrate enhanced anti-PD-1-mediated tumor-cell killing, and single-cell RNA sequencing identified expansion of GNLY^+^CD8^+^ T cells. Pharmacological TLR5 inhibition attenuated NF-κB activation and this cellular response (Zhang et al., 2025).

Protective effects may emerge with reduced inflammatory input. A *Parabacteroides distasonis* membrane fraction suppressed LPS-induced TLR4 reporter activity and MyD88–AKT signaling in CRC cells. Dietary lyophilized *P. distasonis* reduced tumors in high-fat-diet-fed, AOM-treated mice and lowered colonic MyD88 and AKT activation (Koh et al., 2018). *Lacticaseibacillus rhamnosus* LS8 reduced AOM/DSS-associated tumorigenesis, which was accompanied by lower serum LPS levels, reduced colonic TLR4 and NF-κB expression, and less barrier injury (Wang et al., 2022).

Bacterial combinations also show potent benefit against CRC development through TLR signaling. In AOM/DSS-treated mice, a mixture of *Lactobacillus johnsonii* and *Limosilactobacillus reuteri* reduced tumor burden in wild-type but not *Tlr6*-deficient mice. It is associated with inhibition of mucosal inflammation and a reduced abundance of Proteobacteria (Kim et al., 2020). Administration of a probiotic cocktail containing *Lactobacillus acidophilus, Bifidobacterium bifidum, and Bifidobacterium infantum* with oligofructose and maltodextrin reduces colon cancer development in a rat model. This effect was associated with improved gut barrier integrity mediated by TLR2 and suppression of apoptosis and inflammation (Kuugbee et al., 2016).

## 5 Discussion and future perspectives

The available evidence does not support classifying TLR signaling in CRC as uniformly tumor-promoting or tumor-suppressive. Similar variability in TLR function has been described in other cancers (Hoden et al., 2022; Janjirala et al., 2026). A convincing mechanism would show that a bacterium or its product reaches the relevant tissue compartment, engages a particular TLR in an identified host cell and produces a CRC-relevant phenotype. In addition, its biological significance may vary with microbial strain and ligand structure, the surrounding community, the state of the responding cell, and can be affected by disease setting, and the timing and duration of exposure.

Species-level classification is often too coarse to explain bacterial–TLR interactions in CRC, as seen in the contrasting NTBF findings discussed above (Lee et al., 2018, 2021; Kordahi et al., 2021). A phenotype observed with one isolate in one model may not represent the species as a whole. A broader screen of 277 human gut isolates found that TLR-dependent innate responses can vary among strains (Spindler et al., 2022). Strain identity alone, however, may not fully explain the pro-tumor or anti-tumor effect. In many studies, the microbial component responsible for TLR activation remains undefined. Even when a candidate ligand is implicated, a broad designation such as LPS may not provide sufficient mechanistic resolution. LPS preparations from different CRC-associated bacterial species can elicit distinct cytokine responses (Sulit et al., 2023). LPS from *Fusobacterium animalis* ATCC 51191 also bound Siglec-7, and structural analysis of *Fusobacterium polymorphum* ATCC 10953 identified a Siglec-7-binding epitope within the LPS O-antigen (Lamprinaki et al., 2021; Di Carluccio et al., 2025). An LPS-induced phenotype may therefore involve receptors other than TLR4. Conversely, TLR4-dependent signaling can be initiated by host-derived damage-associated molecular patterns (DAMPs), such as HMGB1 released from dying tumor cells (Apetoh et al., 2007).

A microbial product can affect CRC through a TLR only if it reaches a cell that expresses the receptor. Its anatomical distribution and the identity of the responding cell are therefore important when evaluating the proposed mechanism. Recent spatial methods now permit host and microbial signals to be examined within the same tissue section. Spatial imaging of human CRC and paired adjacent tissue revealed regional LPS accumulation and colocalization with distinct immune-cell subsets, although the bacterial source of the LPS and its relationship to TLR4 activation were not determined (Walberg et al., 2026). In chronically stressed tumor-bearing mice, *Enterococcus gallinarum* translocated from the gut to tumors, where its phage DNA activated TLR9 in cancer-associated fibroblasts. Fibroblast-derived glucocorticoids then impaired antitumor germinal-center B-cell responses (Bashir et al., 2026). Future studies should test whether a given microbial product reaches a receptor-bearing cell and activates the proposed TLR pathway within the same tissue niche. Colocalization would support spatial plausibility. Testing causality, however may require complementary perturbation of the ligand and receptor in the relevant cell population and tissue site.

Bacterial load has received limited attention in mechanistic studies of bacteria–TLR signaling in CRC. Many experimental studies test a single exposure level, while sequencing-based human studies often provide compositional data rather than measurements of absolute bacterial abundance. Dose–response experiments could quantify the burden achieved in mucosal or tumor tissue and assess TLR pathway activity and CRC phenotypes in the same model. Such studies could reveal dose-dependent or threshold effects that single-exposure designs cannot detect.

Temporal context remains insufficiently resolved in studies of bacteria–TLR signaling during CRC development. Many carcinogenesis models begin exposure to a bacterium or microbial product before or during tumor induction and continue it through tumor development. Constitutive receptor-deficient models cannot determine when receptor signaling matters because the receptor is absent throughout disease development. Cell-culture experiments generally examine acute responses in established CRC cells. Introducing and withdrawing a defined strain or product at different stages, together with inducible TLR perturbation in selected cell populations, could help distinguish effects on tumor initiation, progression and treatment response and reveal whether they require continued microbial exposure.

Another question is whether bacterial exposure leaves a durable host-cell state. A recent study provides a relevant precedent. Repeated DSS-induced colitis produced a clonally inherited, AP-1-associated chromatin memory in colonic stem cells that persisted for more than 100 days after inflammation resolved and enhanced tumor outgrowth following *Apc* loss (Nagaraja et al., 2026). It would be interesting to explore if sustained or recurrent TLR activation by bacterial ligands could produce a comparable form of memory. Lineage tracing combined with single-cell transcriptomic and chromatin profiling could determine whether such reprogramming persists in epithelial, immune or stromal cells and changes subsequent tumor development or treatment response.

Therapeutic translation exposes the limitations of assigning a fixed function to individual TLRs. TLR4-dependent responses in CRC cells and macrophages have been linked to reduced sensitivity to cytotoxic chemotherapy (Yu et al., 2017; Fragkoulis et al., 2026). Other microbial products acting through TLR2 or TLR5 have shown antitumor activity in preclinical models (Kang et al., 2023; Silva de Oliveira et al., 2025). In an MC38 model, microbiota-derived hexa-acylated LPS enhanced anti-PD-1 activity. In a separate experiment, TLR4 inhibition during anti-PD-1 treatment substantially reduced treatment efficacy (Sardar et al., 2025). These findings involve different ligands, cellular compartments and treatment settings, so they do not demonstrate opposing effects within a single model. They nevertheless caution against treating a TLR as uniformly favorable or unfavorable. Interventions directed at a defined microbial product or responding cell population may offer greater precision than broad receptor agonism or blockade.

Human CRC studies support several candidate bacteria–TLR interactions, but their activity within specific tumor compartments and their contribution to disease course or treatment response remain uncertain. Most mechanistic evidence comes from AOM/DSS experiments, genetic mouse models, transplantable tumors and cell-culture systems, each representing a different aspect of the disease. Prospective studies could examine selected bacteria and their products alongside associated host-cell responses in biopsies or surgical specimens. Clinical follow-up would help determine whether these findings are associated with progression, recurrence or treatment response. Patient-derived organoids cocultured with immune or stromal cells, or tumor explants, can be used to test whether these responses depend on the implicated microbial product and TLR. With sufficient experimental support and safety data, controlled clinical studies can then be designed to assess whether interventions targeting specific bacteria–TLR interactions improve treatment response or reduce recurrence in patients with CRC.
